# Digital twin-driven strategic demolition plan for circular asset management of bridge infrastructures

**DOI:** 10.1038/s41598-025-94117-8

**Published:** 2025-03-27

**Authors:** Sakdirat Kaewunruen, Connor O’Neill, Pasakorn Sengsri

**Affiliations:** 1 Department of Civil Engineering, School of Engineering, University of Birmingham, Edgbaston, B152TT UK; 2 Digital Asset Management, AtkinsRéalis, Birmingham, UK; 3 Department of Highways, Bangkok, Thailand

**Keywords:** Digital twin (DT), Building information model (BIM), Infrastructure asset lifecycle, Demolition, Circular economy, Engineering, Civil engineering, Mechanical engineering

## Abstract

Economic growth plays an important role in the rapid increase in construction of transportation and bridge infrastructures, which in turn causes enormous greenhouse gas emissions contributing directly to climate change. An innovative and effective method, so-called Building Information Modeling (BIM), to sustainably manage detailed lifecycle of infrastructures, has been recently adopted to revolutionise the Architecture, Engineering and Construction (AEC) industry. Its major function is to sustainably optimise all detailed stages of an infrastructure asset’s lifecycle. A three-dimensional architectural BIM incorporating three additional dimensions (time, cost, and carbon emissions) has then been created in this study to virtualise the whole lifecycle performance of bridge infrastructure through BIM data. For circular asset management, multi-scale details of assets and infrastructures are indispensable. On this ground, these information dimensions are highly critical to asset managers to assure not only public safety, but also sustainability over the whole lifecycle. It is thus critical to quantify carbon footprint in order to identify better alternative solutions for construction and maintenance, resulting in carbon neutrality and carbon credit. Our digital twin (DT), driven by the BIM, has embedded demolition scenarios whose lifecycle cost and carbon footprint can be quantified and optimized simultaneously. Our study is the first to also demonstrate circular end-of-life management through strategic demolition planning that enhances circular economy practice. This aspect is novel and has not been commonly adopted in practice. Our study reveals that the construction stage of the asset lifecycle for this study is the main contributor to carbon emissions and costs stemming from raw materials and their productions. This eventually leads to significant waste at the end of asset’s life, requiring strategic demolition plan to maximise reuse, repurpose, and recycle of materials, parts and components. Our innovative DT is capable of dealing with the cradle-to-cradle lifecycle management. Another co-benefit of using the BIM-based digital twin is to minimise streamlining design, re-work, mitigating risk, and real time processing of design changes in all stakeholders, reducing the effect on carbon emissions, costs, and time schedules. All dimensions (i.e. 6D) can be updated and re-calculated in real time when cross-linked with inspections and condition monitoring, generating real-time digital twin driven solutions.

## Introduction

In recent years, one of the most pressing concerns of bridge infrastructures is how well the bridge maintenance is being managed^1^. A dramatic increase in bridges’ construction due to increasing demand for transport infrastructures in developing countries is likely to affect the total environment, since it requires swiftly more raw materials. This will increasingly emit high amounts of carbon dioxide and other greenhouse gases (GHG), which fundamentally contribute towards global warming and climate change effects. Based on lifecycle perspective, the construction stage is the most dominant stage contributing to GHG emissions over asset lifecycle^2^. However, it is also crucial that the maintenance activities and end of life management are traceable throughout different stages of asset life. Traceability will enable adequate and optimal circular practices of the asset and its components. To enable circular asset management, full details of materials, components, parts and assets are indispensable. In practice, whole life carbon emissions can be thoroughly quantified by using the life cycle assessment (LCA)^3^. This approach involves the determination of cost and carbon footprints over the whole lifecycle of any class of asset^3^. Considering the plethora of materials, suppliers, stakeholders, contractors, energy sources, and industry standards, conducting an LCA of an infrastructure is significantly more complex than that of a manufactured product^4^. Using traditional software and tools for LCA of complex buildings can cause either excessive over- or under-estimation. This can be because various asset components may have different lifetimes, and many assumptions made in LCA software cannot adequately respond to complexity and locality perspectives (e.g. time-dependent variations in expected rate of returns, asset maintenance activities, novel replacement materials, component dimensions, constant change in asset innovation during operational stage, recycling technologies, etc.). On this ground, the lifecycle project costs and delivery timelines including the improved quality of complex infrastructures should be better conducted by using Building Information Modelling (BIM) with an integration of the infrastructure LCA^5^. BIM has been initially developed as a technique to improve constructability, but has transformed over time into a process to manage assets through construction, to maintenance and to end of life management.

Building information modelling (BIM) is a new process and tool to digitalise engineering assets and upkeep the digital asset information, which has been adopted in construction industry. It is a three-dimensional digital replica of asset(s) such as buildings and bridges, which contain complex architectural information and asset details (e.g. dimensions, materials, parts, and components). BIM has evolved from traditional 2D CAD models (or blueprints) to 3D CAD models embedded with multi-purpose information layers (e.g., construction time sequence or 4D-BIM), improving information sharing and asset traceability in construction. BIM has now been essential in various countries; for example, new UK BIM standards require asset owners to keep and maintain building information. In recent years, BIM has incorporated an interoperable concept that can enhance the whole life-cycle assessment (LCA) and circularity of the built environments. BIM can be further extended to embed six dimensions (6D) of information layers where time sequence, cost and carbon impacts can now be quantified, visualised, and reported in real time. These attributes can strategically help stakeholders to monitor, optimise, and reduce any unexpected consumption and waste throughout the whole life of the assets (e.g. from cradle to grave, and/or from cradle to cradle). The 6D BIM can be enhanced towards a digital twin (DT) through collaborative NavisWork and/or Domino that provides real-time interactive crossover workflow between 6D-BIM and LCA.

Lifecycle stages of products, components and/or assets traditionally include (i) raw material extraction, also called the “cradle”; (ii) manufacturing & processing; (iii) transportation; (iv) usage & operation; (v) waste disposal, also called the “grave”. There are 3 main product lifecycle models implemented in LCA:

- Cradle-to-gate: this model only assesses a product’s footprint until it leaves the manufacturing gates before it is transported to the consumer (stages i and ii).
- Gradle-to-grave: this model includes all 5 lifecycle stages in the footprint measurements or LCA. It shows a full footprint from start to end. This is the standard lifecycle model in the current “linear” (as opposed to circular) economy.
- Cradle-to-cradle: this model is closing the circular loop by replacing the waste stage at the end of life with either downcycling or upcycling process that makes it reusable or recyclable for another product or project. This model will create a closing loop of ‘make, use, and reuse’ in order to create a regenerative model of resources, to minimise wastes, and to reduce the extraction of new raw materials/resources. This is the fundamental for circular economy.

In this study, we will investigate sustainable lifecycle assessment from cradle to cradle of the bridge infrastructure using BIM method and establish the digital twin (DT) for the demolition planning for practical circular strategies. To the best of our knowledge following a critical review of the available literature, there are no current works focusing on the improvement of the cradle-to-cradle lifecycle sustainability of a bridge infrastructure, using DT technology. It is important to note that federated digital twin (i.e. high-level data representative) is utterly inadequate for efficient asset maintenance and operations since details of materials, components, assets and localised conditions must be comprehensively recorded. BIM-based DT technology is therefore more suitable to circular asset management. We have chosen a standard road bridge commonly used in Thailand to demonstrate the practical application. Although this roadway bridge is standardized and commonly adopted in Thailand, it has unique construction sequences that can accelerate the construction speed. The precast components of the bridge are modular and can be rapidly assembled on site. This modularity enriches the potential to automate circular end-of-life management of the bridge, which is the main goal of our study. The findings can be used for quantifying the sustainability benefits for similar bridges, resulting in the possibility of predicting the cradle-to-cradle lifecycle of various types of other bridges. It is clear that the potential of asset lifecycle analysis on the whole bridge infrastructure can be affected by the BIM. Through three-dimensional design, a BIM model can further consist of six dimensions, including cost schedule, carbon footprint, and time schedule information. Real time interaction between BIM and LCA to enrich DT can be implemented using NavisWork. Additional dimensions to update a digital twin (DT) model are also be implemented over the asset lifecycle. This study aims to generate a digital twin that differs from a conventional 6D BIM model through the use of connected mathematical models that will automatically update when a model value is changed, providing a cyclical output through real time processing^6^. The DT model can provide real-time information for end-of-life management (i.e. demolition planning and component traceability) that will enhance asset circularity. In this paper, the following section will describe an overview of bridge infrastructure (chosen for the demonstration), followed by the literature review and methods sections. Lastly, the results, discussions, and application sections will be presented.

## Background of use-case Bridge infrastructure

In this paper, a standard roadway slab bridge^7^ has been selected for demonstration. A BIM model has been established for bridge infrastructure and verified using design and construction data by industry expert engineers of the Department of Highway. This bridge model basically includes a width of 9 m, a total span of 27 m and a 8-m height over the ground level. The BIM has adopted industry design information of traffic volume, maintenance activities, generic geometry design, transit distance for raw materials, and lastly service life. Considering materials transportation, the raw materials are transferred to the construction area with an average of 50 km. An average of 60 km/h traffic flows are set for the service of this designed bridge. Accordingly, the bridge infrastructure has a short design service life of 30 years since it is affected by the high volume of traffic^8^. General maintenance requirements of the bridge infrastructure include drainage clearing, bridge cleaning, and routine inspection.

In terms of materials costs (considering locality in rural Thailand), the total cost estimation of these works is expected to be an average cost of £300 per year. For other minor maintenance, for example, bridge repair and pavement replacement are performed with an average materials cost of £5,000 every 10 years. Further assumption is the construction schedule of the particular bridge infrastructure that cannot be found in common asset lifecycle standards. Thus, this can be estimated through literature^8^. Our LCA model has adopted these assumptions and information. The LCA model has been developed in Excel and simultaneously co-simulated with BIM to generate a DT using NavisWork.

## Literature review

### Digital twin (DT) & Building information modelling (BIM)

According to a review^6^, Digital Twin (DT) is a term that does not have a specific and unique definition, from literature it can be seen that the concept originated in an aerospace field but has now been adopted into the infrastructure domain. However, digital twin can be generally defined as a virtual representation of a product, characterized by synchronization between the virtual system and the real system, through detected data (from inspections and/or sensors) and connected intelligent devices, mathematical models and real-time data processing^9^. In essence, DT is a computer model, which can mirror and simulate any asset and the environment that the asset is in within a defined system boundary. Digital Twin has the capabilities to improve decision making and further understand the effects of making design changes through data processing. In general, the main functions of DT are to simulate, predict, monitor, analyse, and control the formation processing and response of physical products in the real environment conditions^10^.

Building Information Management (BIM) has been demonstrated to have a very diverse set of definitions from various sources of literature^11^. Basically, a BIM model is considered as a shared digital model of a built asset to facilitate design, construction, and operation processes to make a reliable basis for decisions^12^. Current BIM concept and applications provide static dimensions of information. However, in recent years, BIM has enabled an opportunity to further construct a digital twin (DT) where 3D architectural model in BIM can be routinely updated by condition monitoring via (i) real time sensors or (ii) routine inspection. The dynamic BIM will result in a dynamic update of information (e.g. dimensions, costs, carbon footprint, time sequence, etc.), and becoming a DT of the assets. Thus, it is necessary to deeply understand BIM’s concepts and capabilities that can be applicable to practical problems. In this study, we will demonstrate the transformation of a traditional 3D BIM onto a DT or a ‘dynamic BIM’ with a ‘living’ characteristic of information flows and updating. The application is relatively new in the industry and the demonstration can help engineers to adapt and evolve the traditional BIM through real time data^13^.

Digital twin (DT) has already been proven to be highly beneficial, due to the embedment of data in 3D CAD models, rendering the final model 4D, 5D or in the case of Digital Twin, 6D or more. Note that the 3D dimensions of assets can change over time (e.g. by corrosion, by retrofitting, by alternative material replacement, or by structural modification). These changes can result in the change of other key dimensions of information. The other key dimensions include the material costs, lifecycle parameters, construction times, and carbon footprints. It is noted that DT can also give significant benefits through the combinations of risk identifications and cost efficiency of projects. This allows many parties and stakeholders involved to cooperate for infrastructure construction projects. In the past, many designers have created building designs in a reduced timeframe and enabled on-site laborers to minimise mistakes, and thus decreasing re-work or even maintenance activities^14^. However, due to over 80% of existing buildings in Europe built by 1990, it is difficult to derive their BIM documentation, resulting in the difficulty of generating a DT or BIM models^15^. Table 1 summarises the levels of BIM maturity and the classification at each level, which describes the transformation of BIM-based technology. This illustrates the transformation of BIM across scales towards digital twins in practice.

**Table 1:** Transformation of BIM maturity.

Classification	Definition	Transformation of capabilities and added values
Level 0	Computer Aided Design (CAD)	Technical drawings, Hand drafts/sketches, Unorganized CAD, 2D data sharing and/or exchange
Level 1	CAD, 3D model	Organized 2D CAD and/or 3D architectural model with a participative platform, enabling a unified data exchange with a standard methodology for data structure and format.
Level 2	Building Information Modelling (BIM)	An organised 3D architectural model on a virtual platform using a software for data sharing and exchange; any sensitive data will be privately managed by specific software or platform and later integrated to the main BIM system by proprietary interfaces or special middleware. The BIM can further add more dimensions of data or information. This can include 4D for construction sequencing (time dependent), and 5D for financial transaction or cost details with respect to the time sequence.
Level 3	Digital Twins (DT)	This level includes a fully integrated and participative platform that can be interconnnected to oher system via internet or an interactive middleware. ‘Web service’ can be adopted to activate the workflow for information network. This can be done via intranet, cloud, co-simulation, or Navisworks, etc. Industry foundation class standards must be complied with. In principle, minimum 6 dimensions (6D) of information should be embraced. The DT must include physical dimensions in 3D (width, length, depth), and other critical dimensions of information such as construction sequencing (4D), financial and cost item (5D), project contracts or legal arrangement (6D), and carbon footprint, environmental impacts and toxicity information (7D).
Level 4	Digital Twins (DT)	To achieve this level, the DT must incorporate live data (e.g. regular inspection, or condition monitoring data). These can be derived from various sensors (live actions, burden responses, ambient conditions, dwelling level or human response). This can also include facility management.
Level 5	Digital Twins (DT)	This is an automated level for DT when a decision making can be either fully or partly automated from data-driven approaches or physics-based simulations. The DT will be interconnected with advanced data fusion (by using multi-source data from inspections, real-time sensors), and predictive co-simulations (through either empirical and constitutive models, analytical and numerical methods, artificial intelligence and machine learning, or other emerging techniques, etc.).

### BIM implementation with sustainable lifecycle management

**Fig. 1:**
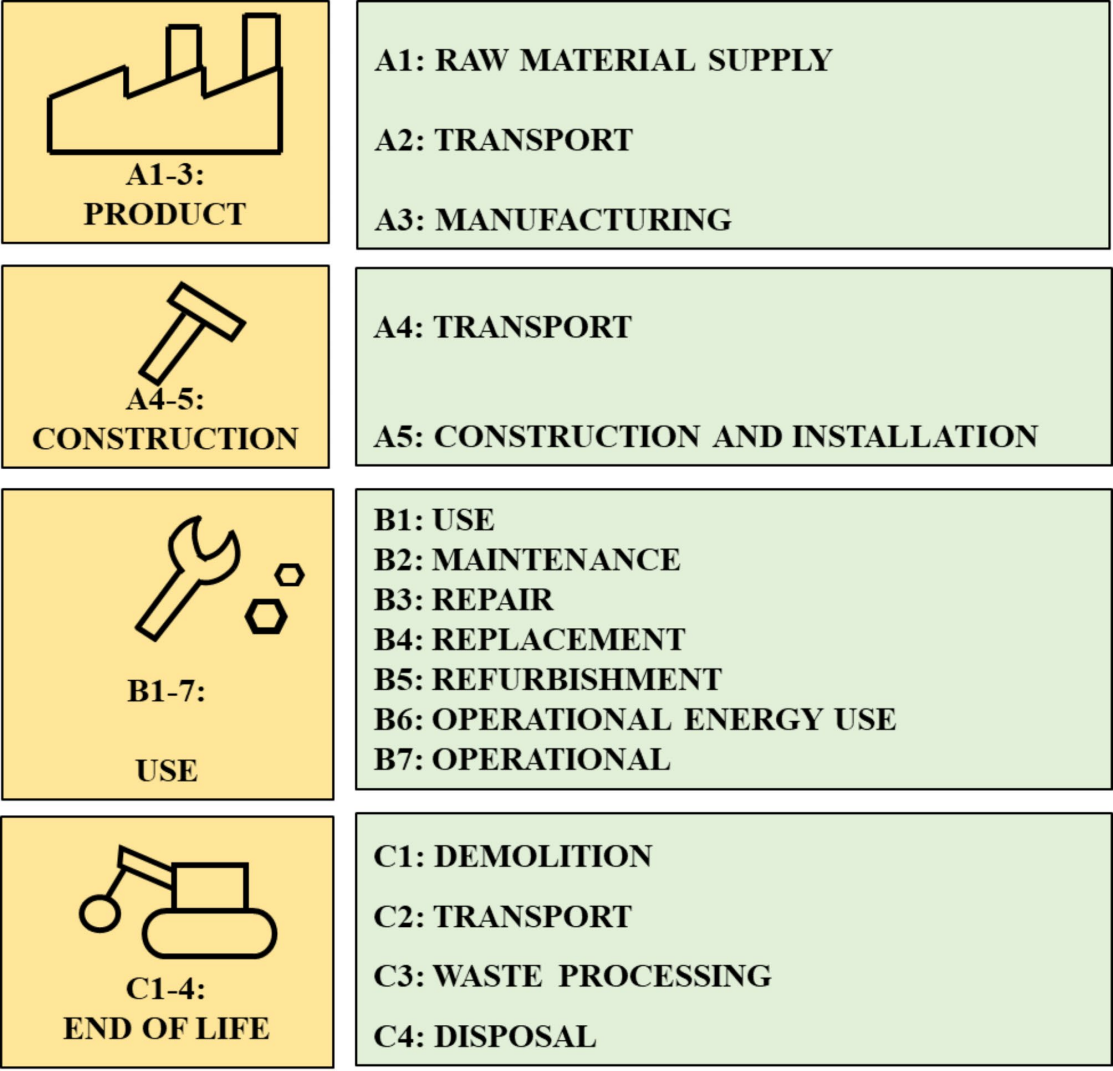
LCA Scope in typical infrastructure lifecycle.

**Fig. 2:**
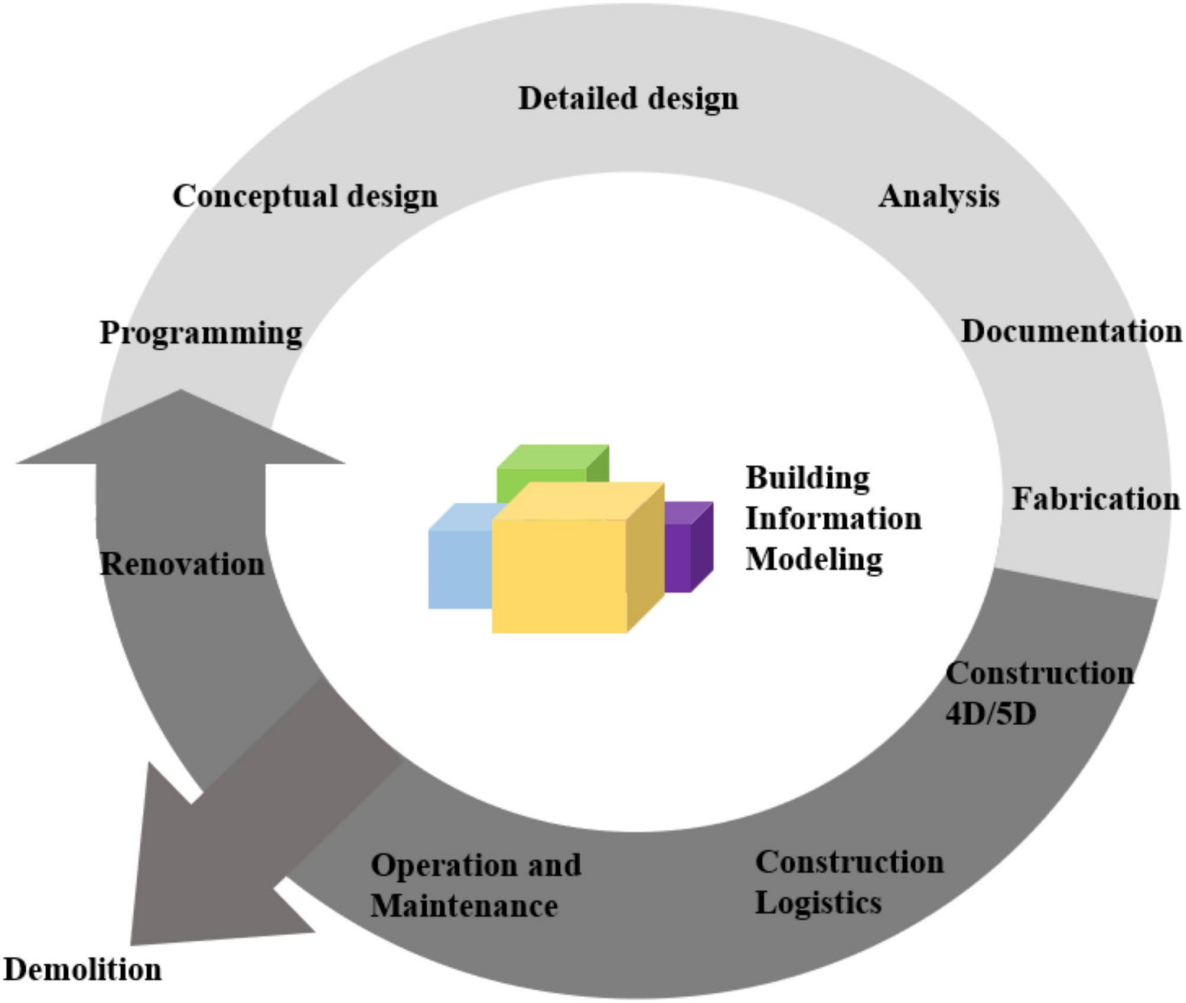
BIM LCA scope in typical infrastructure lifecycle.

A life cycle assessment has been employed to assess and understand the sustainability of a certain piece of infrastructure over its entire lifecycle^16^. A good representation of the stages involved during a typical lifecycle for generic infrastructure can be seen in Fig. 1 and a BIM specific life cycle for generic infrastructure in Fig. 2.

There is currently a great interest in the use of Digital Twin and BIM Technology for assessment of the environmental impact of infrastructure throughout its lifecycle. The use of this technology can be highly beneficial in the early stages of asset life, particularly in the design phase^19^. For new infrastructure projects, there has been recognised potential for its benefits towards sustainable design^20^. This has been confirmed by the review of many different existing research work, which focuses on how BIM can monitor environmental sustainability and manage buildings throughout their life cycle. Currently there is no perfect method of integrating BIM with lifecycle management, however a conceptual framework devised by Ma et al.^21^ shows how BIM can be implemented into the asset lifecycle management to ensure that all conceptual benefits are achieved. Examples of using this method are prevalent during the design and pre-construction stage. BIM has been found to be an effective method for quantifying and measuring carbon emissions. A BIM approach to lifecycle management can help reduce carbon emissions by providing more accurate quantitative production and thus less generated waste during the projects’ preconstruction phase^22^. The material production phase accounts for 93.4% of all CO_2_ emissions used from the design phase up until the final construction of the asset^23^. This stage of the asset lifecycle is particularly influential on the total carbon footprint of the assessment. Developing a BIM model into a digital twin (DT) can allow the users to quickly change components and/or materials during the design phase, in order to observe the effects on the asset’s carbon footprint, from pre-construction phase, all the way to the construction phase, taking into account raw material production and material transportation^24^. This therefore showcases the capability of BIM since materials can easily be compared and contrasted, ensuring that the optimal material or component is chosen. Despite considerably less literature surrounding Digital Twin, as mentioned earlier, BIM and Digital Twin share similar concepts, and therefore the benefits recognised with BIM utilisation for lifecycle management can also be considered for Digital Twin, which will only enhance these benefits through automatic real time data processing and monitoring. For these benefits to be realized, the Digital Twin requires relevant stakeholders’ input. This is certainly a limiting factor because the lack of client demand, data and senior support, and DT knowledge can result in sub-standard lifecycle management^24^.

With respect to later stages of asset life cycle, there is considerably lesser literature published, however a good example of how cost estimates and carbon emissions are analysed can be found in a series of studies from Kaewunruen et al.^8,25^. These studies demonstrate an effective method and guidance for multi-dimensional BIM and Digital Twin adoption with lifecycle management implementation. Despite the benefits found by Verdaguer^17,18^, there are serious limitations of using this method that can be achieved for preconstructed buildings, a complete and accurate BIM for old buildings can be unfeasible in some instances^26^. As a result, early stages of the asset life cycle will be impossible to extract any benefit from as they have now elapsed these critical stages. Integrating the BIM software for preconstructed buildings is also very difficult and may not be viable for older infrastructures and therefore resulting in very limited benefits in practice^17,18^.

### BIM application to Bridge infrastructure

The use of the BIM method offers more benefits compared to traditional 2D drafting and design. 2D drawing methods can be very time consuming and prone to errors. On the other hand, DT can negate the need for re-working, data re-entry and re-design, therefore improving reliability of design and shortening time schedules. These benefits have been predominately recognised for newly-constructed building infrastructures and also the DT technologies can be used to enable optimal applications to bridge infrastructures. However, since predictive and preventative maintenance is being adopted as an essential strategy in the infrastructure industry, there is very much a need for a proactive approach for optimised bridge maintenance and renewal^11,27^. In practice, industry design information and performance data can be collectively stored in one common area/ file and can therefore be shared and edited by multiple different parties with ease. This is also beneficial when using the BIM during all stages of asset lifecycle^28^. Further benefits can be recognised when a BIM model has further embedded more information dimensions such as cost, carbon emission and time management that are able to synchronise together so that when a value is changed, all other values are automatically updated to represent the respective change in real time. This enables the BIM model to resemble a Digital Twin of the specific bridge infrastructure.

A 5-dimensional approach using BIM approach has been proven to be effective for both cost and time estimations for a specific case study^14^. This approach would allow for more efficient and streamlined design, resulting in both a more cost and time efficient design. Further benefits of BIM modelling can be obtained through risk management of bridge infrastructures. The early critical stages of the asset lifecycle are where a lot of risk can be identified, analysed and therefore fundamental decisions can be made to minimise any identified risk^29^. However, emerging risks at other lifecycle stages have not been well integrated in BIMs. As a result, various BIMs have become a static information sharing platform, so-called ‘*digital shadow’*, and have not been transformed into a DT.

Despite the huge potential of the application of DT for bridge infrastructures, the data requirements on a very large scale may present challenges obtaining all required industry real-scale data for effective output. A further barrier identified by multiple sources of literature is that there are still large implications and barriers for widespread use of these digitalisation methods in practice^28^. The most notable one is the initial cost of implementing BIM and Digital Twin technology, whether that could be related to software purchasing or training of staff^24,30^.

## Methodology

In this paper, the aim is to develop a novel digital twin (DT) based on 6D-BIM for the effective sustainable lifecycle management of a bridge infrastructure considering diverse demolition options at the end of life. The use case is based on a standard 9 m roadway bridge. The DT will enable end-of-life management towards circular economy.

In this paper, a 3D BIM model has been first generated using Revit 2022 Software. This model contains the digital information and materials database derived from the industry partner. Then, the construction and maintenance data are developed for the standard 9 m roadway bridge throughout the lifecycle assessment, in order to produce a further 3 dimensions, for example, time schedule, cost schedule and carbon emission schedule. The drawback of using this approach is that the software used to develop the information model is tailored only for building infrastructures, however the methodology used in this paper will adjust parts of the software in order to properly model bridge infrastructure construction.

### BIM modelling

Autodesk Revit (2022 version) is used to generate a lean 3D BIM model of the bridge. This model is based on the technical 2D drawing for a standard 9 m roadway bridge^7^. Due to the relative simplicity and symmetry of the bridge used in this paper, it is possible to group together certain components of the bridge with the exact same data and dimensions as presented in the 2D model. These subgroups can then be assembled together in order to create the final bridge structure model. The 3D model can then have all rebars added where are required, as dictated in the technical drawings. After that, material information is added to the whole assembled model, including current market price of the materials and components. The final 3D BIM model is then exported into Navisworks 2020 in order to add the additional 3 dimensions and to transform the model into a rudimentary digital twin. The 4th dimension refers to a construction schedule, resulting from the ‘Time Liner Workflow’ function on Navisworks, and all relevant guidance provided by Navisworks is used to develop this data. Cost data used as the 5th dimension is added by using Revit software.

**Fig. 3:**
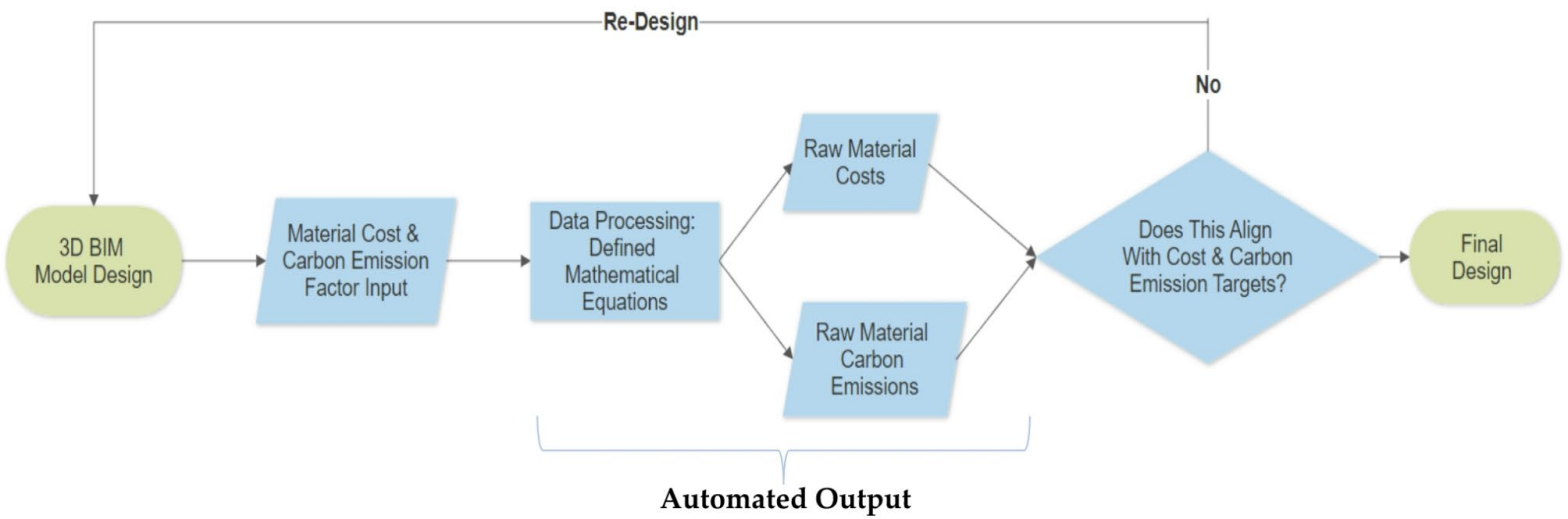
Flow chart of BIM material and cost updating process.

Basically, the cost data is divided up into 2 sections: (i) current market prices of the raw materials and (ii) machinery costs for construction, maintenance and demolition. Both costs are combined to give a cost estimation and a rough cost schedule estimation for all stages of the asset lifecycle. The final dimension of the model defines the carbon emissions, resulting from material volumes, weight and the carbon emission coefficients described in the latter section. Cost and carbon emissions are related to the 3D BIM model through mathematical equations as dimensions change in the BIM model. The updated costs and carbon emissions are processed in real time, forming attributes and features for a digital twin (Fig. 3).

### Lifecycle assessment scope

**Fig. 4:**
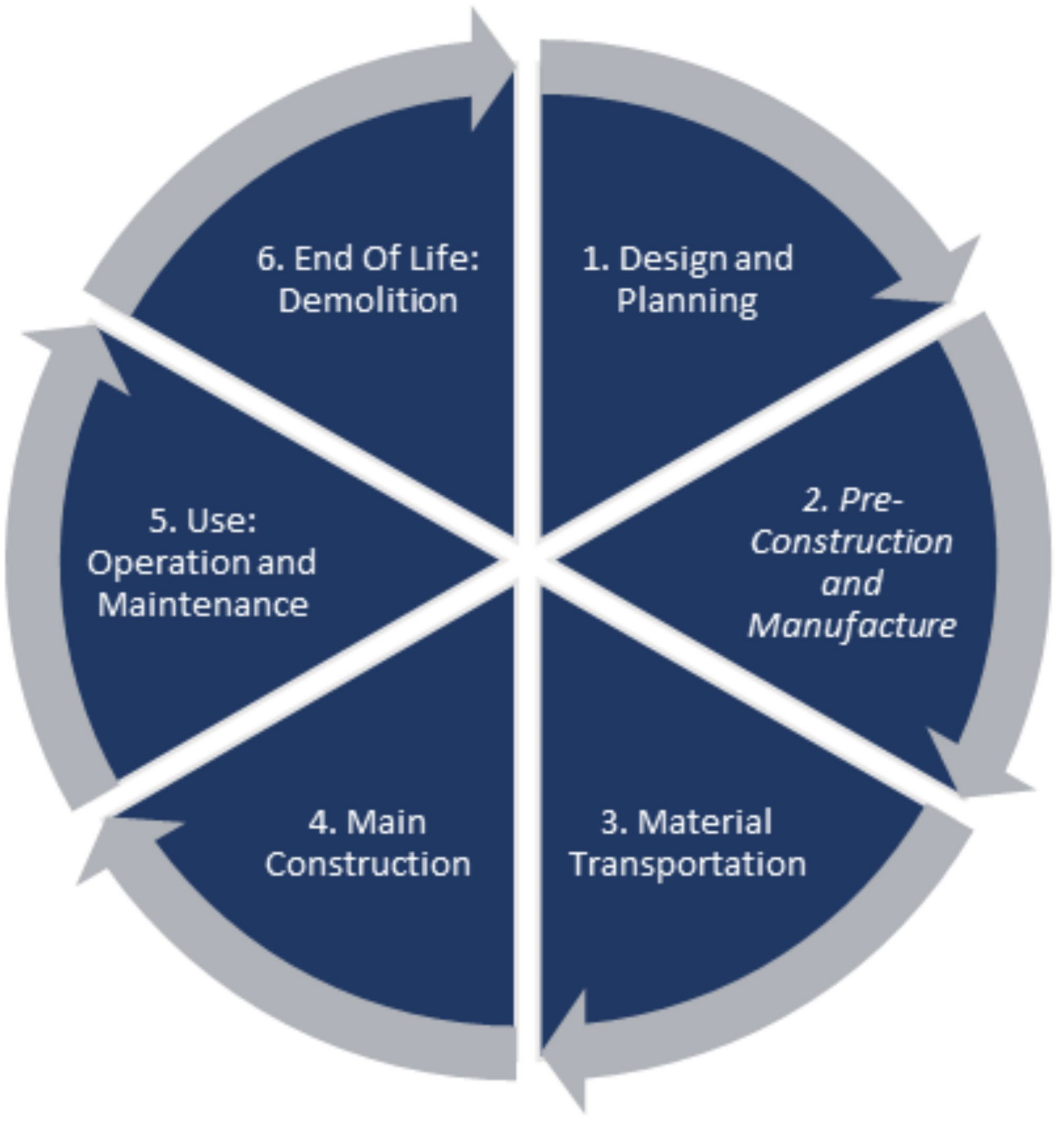
Life cycle assessment scope for a bridge infrastructure.

It is important to scrutinize all stages of an asset’s lifecycle. The improved lifecycle fundamentals presented in Figs. 1 and 2 are considered to create a more specific approach for the bridge lifecycle in this study (see Fig. 4). This approach has taken into account all information discovered via the relevant reviewed literature and is inspired by a new approach to LCA for bridge infrastructure^8^. As illustrated in Fig. 4, this proposed method for LCA will yield higher efficiency and result in improved lifecycle sustainability.

### Data extraction for all phases of lifecycle

As shown in Fig. 4, industry data for all lifecycle stages and predefined asset lifecycle stages are extracted from industry databases and expert advice. This data presents the 6th dimension to the DT, as described in Table 1. In this study, the greenhouse gas conversion factors are based on the green gashouse emission as shown in Table 2. The carbon footprint can then be estimated by using the following equation (see Eq. 1)^31^.

1 $${\text{Greenhouse}}\:{\text{Gas}}\:{\text{Emissions}} = \:{\text{Activity}}\:{\text{Data}}\: \times \:{\text{Emission}}\:{\text{Conversion}}\:{\text{Factor}},$$

**Table 2:** Greenhouse gas conversion factors^31^.

Activity	Unit	Year	Kg CO2e	Kg CO2
Electricity generated	Kwh	2018	0.28307	0.29088
Diesel (100% Mineral)	Litres	2018	2.68779	2.6502
Fuel Oil	Litres	2018	3.17799	3.16633

#### Design and planning

At this early stage in the asset’s lifecycle, there is not a large amount of carbon expenditure. It can be assumed that the greenhouse gas emissions will be determined based on the number of site investigation visits and subsequent fuel consumption.

#### Pre-construction and manufacture

The manufacture of all the raw materials required for construction plays an important role in the carbon emissions for this stage of the lifecycle. Table 3 demonstrates the material emission factors^32,33^. It is important to note that the material costs are based on the current market prices for each material.

**Table 3:** Material C0_2_ factors^32,33^.

Material	Unit	KgC0_2_e
Concrete C30	m^3^	316.8
Concrete C55	m^3^	362.4
Rebar	kg	1.86

#### Material transportation

Material transportation costs are based on types of trucks and the distance between sites at different times. Before the construction stage, rebars are already transferred to the site, but concrete is moved during and throughout the construction phase and waste evacuation is needed to be transported from the site after the construction for waste elimination.

#### Main construction

During this stage, carbon emissions are predominantly released from the machinery required to construct and assemble the bridge. Examples of machinery are welding equipment, crane trucks, drilling machines, rebar cutters, etc.

#### Use phase: operation and maintenance

As mentioned earlier, due to the nature of the asset, the operational aspect of the lifecycle stage is negligible as no significant costs or carbon emissions are produced by the asset during the operation. However, bridge maintenance activities are taken into account for both costs and carbon emissions.

#### End of life: demolition

It is important to consider the demolition stage of the lifecycle. 80% of the demolition materials from a typical construction site are regarded as disposable and the remaining 20% is recyclable material^34^. To estimate the carbon emissions from the demolition stage, it can be calculated using Eq. 2^8^.

2 $${\text{Greenhouse}}\;{\text{Gas}}\;{\text{Emissions}}\;\left( {{\text{GGE}}} \right)\;{\text{in}}\;{\text{demolition}} = {\text{GGE}}\:{\text{in}}\:{\text{Construction}}\:{\text{Stage}} \times 8.95\% ,\:$$

## Results

A six-dimensional BIM of a standard 9 m roadway bridge has been generated, including three additional dimensions (the time schedule, the cost schedule, and the carbon footprint of the bridge). Most informative data is obtained and created using Autodesk Revit and Navisworks 2022, leading to a DT of the bridge. All the results from the BIM-based DT simulations are presented in the following sections.

### Three-dimensional (3D) model of a typical road Bridge

**Fig. 5:**
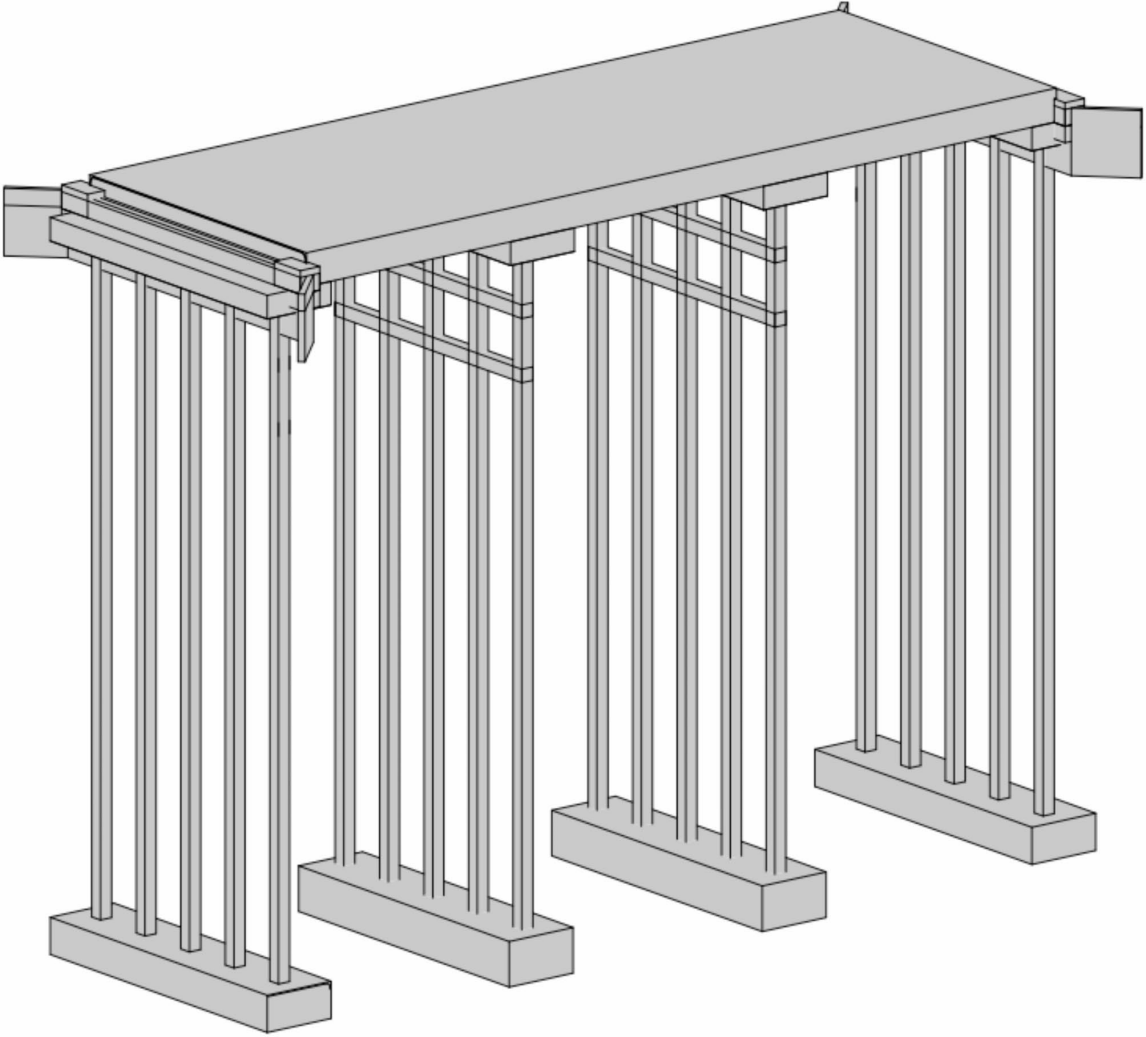
The 3D Model views of 9 m road bridge.

**Fig. 6:**
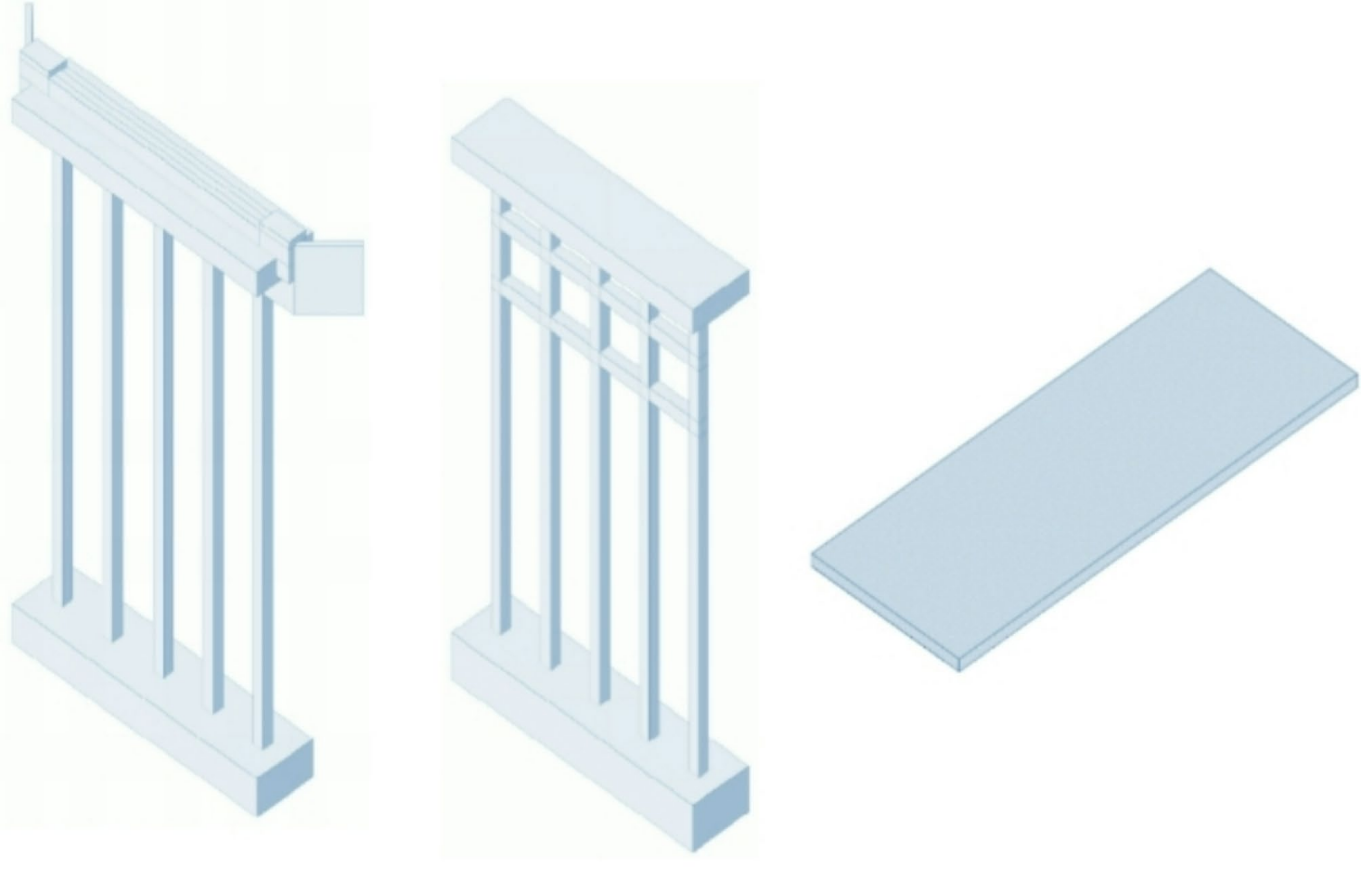
The 3D models of individual bridge components (abutment, pier, and slab deck shown in left, middle, and right side, respectively).

Based on two-dimensional (2D) engineering drawings for a standard 9 m road bridge^7^, a 3D model is comprised of 2D information in the Level of Development (LOD) 300^35^, which is capable of identifying BIM with its specific size, shape, location, orientation and the quantity of each element or component of the bridge. Figure 5 shows the schematic 3D model of the bridge. Basically, the final model including individual components (abutment, pier, and slab deck) is generated using the Revit software (see detailed components in Fig. 6). After that, these components are assembled together to form the final superstructure model.

### Construction schedule & 4D model

**Fig. 7:**
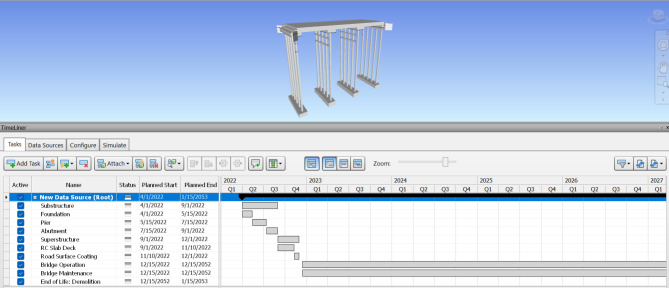
Entire timeline of the bridge in Navisworks 2020.

**Fig. 8:**
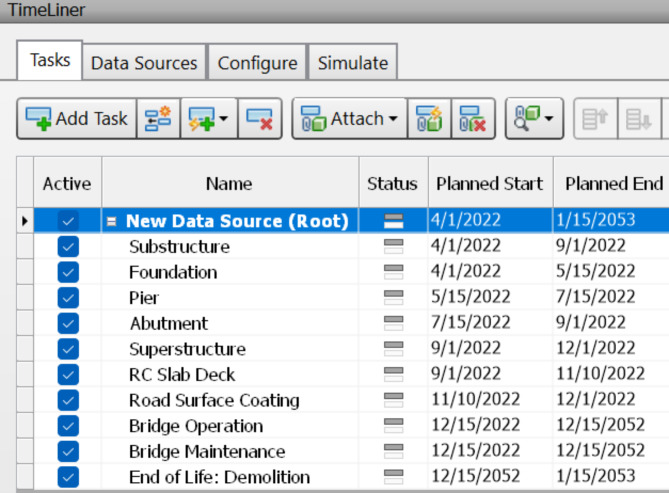
Magnifying timeline of the bridge in Navisworks 2020.

**Fig. 9:**
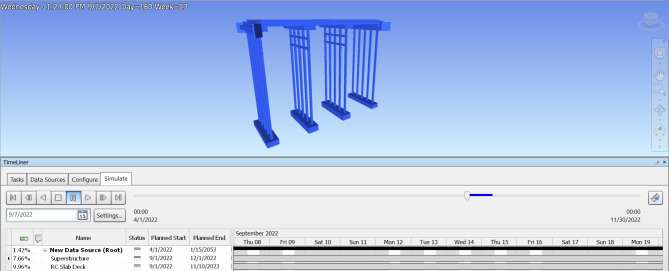
Simulation of slab deck construction.

**Fig. 10:**
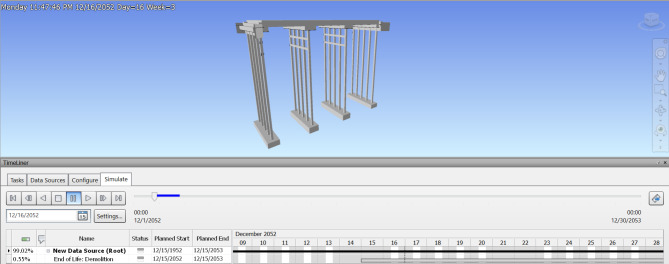
Simulation of bridge demolition.

When a 3D BIM model has been generated, the additional 4th dimension known as a time schedule is added. After extracting the bridge model from Revit software, the time schedule for substructure construction is formed using Navisworks, which offers the capabilities to easily edit the start and end dates, also obtaining its potential demolition date. Figures 7 and 8 show the estimated time schedule from construction through to demolition. Navisworks is also able to use this time data to generate a simulation of the asset life, which exhibits a visual demonstration of how the asset will be assembled by individual component order, along with the demolition of the asset after its full operation period as illustrated in Figs. 9 and 10. The aforementioned time schedule can also provide stakeholders with valuable information and this aspect will be evaluated further in the discussion section.

### Cost schedule & 5D model

**Fig. 11:**
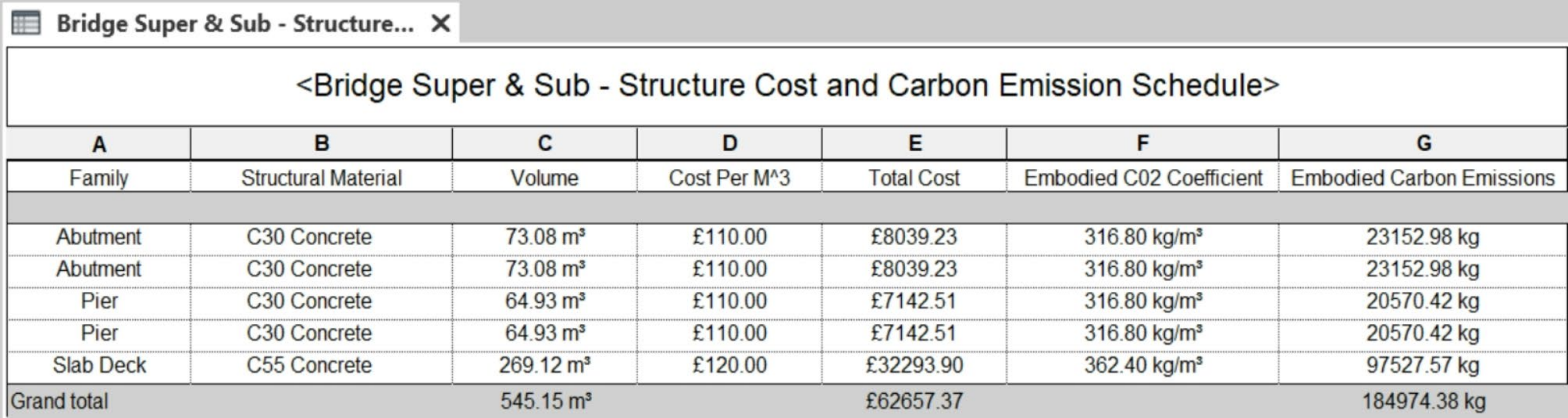
Bridge superstructure, substructure cost and carbon emission schedule in Revit 2022.

**Fig. 12:**
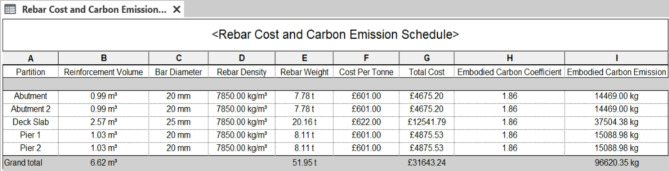
Bridge rebar cost and carbon emissions schedule in Revit 2022.

**Fig. 13:**
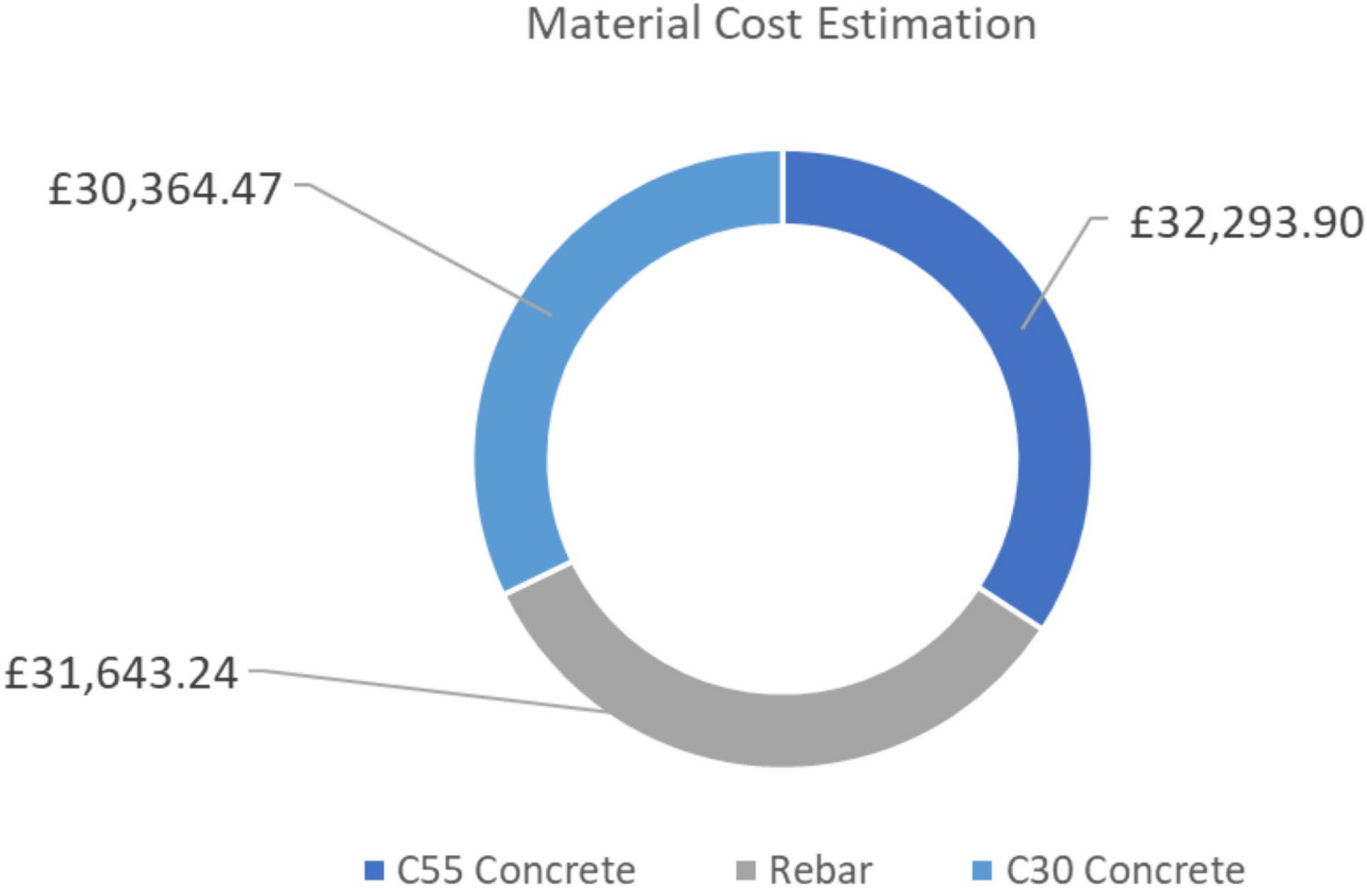
Material cost estimation.

**Fig. 14:**
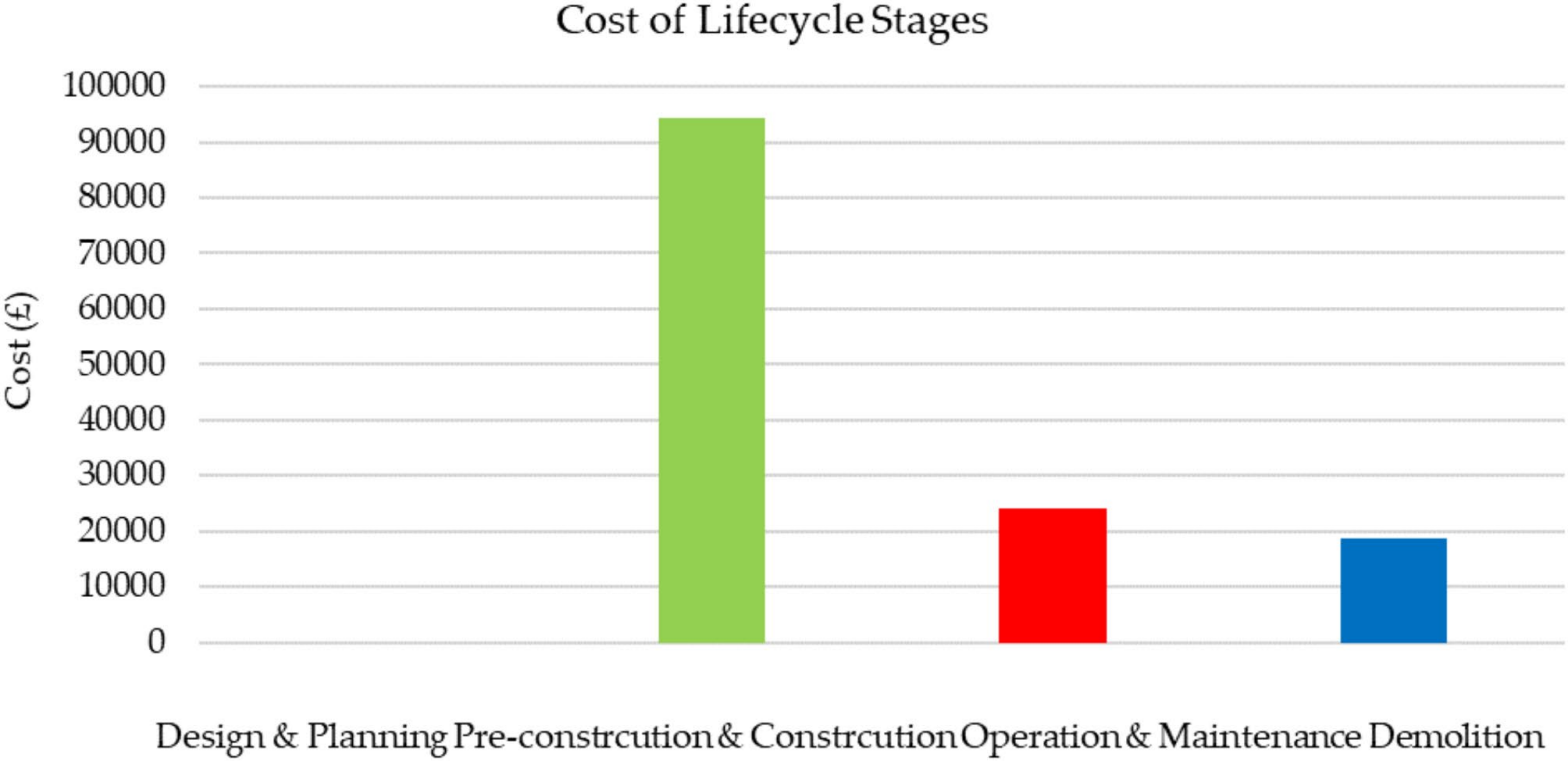
Cost estimations at different stages of asset lifecycle.

In this section, the 5th dimension of the bridge model is the cost schedule. The pre-construction and construction stages of the asset lifecycle play an important role in the total costs. Figures 11 and 12 show a 9 m road bridge’s cost estimation of £30,364, £32,293, and £31,643 for C30 concrete, C55 concrete, and rebars, respectively. Figure 13 illustrates 66.4% of the total raw material cost resulting from concrete only. According to the aforementioned assumptions, the cost of the operation of the asset lifecycle hardly affects the total lifecycle cost of the bridge. Thus, the maintenance cost is calculated for £24,000, as presented in Fig. 14. The final stage of the bridge lifecycle is the demolition, due to no planed budget for this task it is assumed that the demolition cost should be at least 20% of the construction cost for desembing components^8^. As such, the total cost estimation for the entire asset lifecycle of a standard 9 m road bridge is £137,161, which is corresponding to 69% of the cost expended during the pre-construction and construction stages.

### Carbon emissions schedule & 6D model

**Fig. 15:**
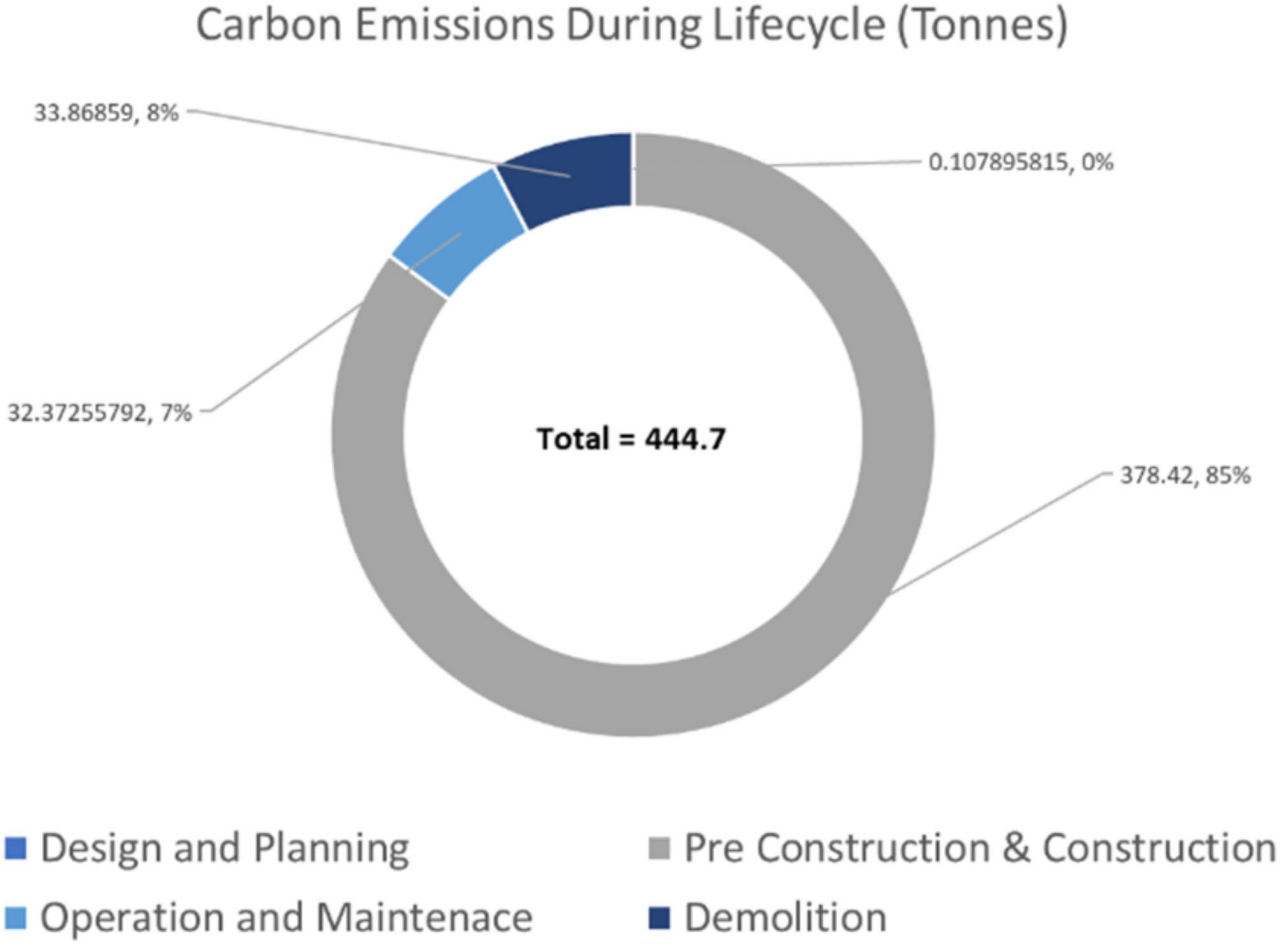
Carbon emissions estimation of materials.

Lastly, the remaining information dimension is added to update the BIM with the carbon emission schedule. For the carbon emission schedule of the raw materials, this is calculated using the CO_2_e factors presented in the methodology Sects^32,33^. These factors are inputted into Revit’s workflow, resulting in total carbon emission calculation for each of the raw materials. Figure 15 shows the total embodied carbon of concrete used in the brige infrastructure, which is 18.5 × 10^4^ kg; and for the rebar is 9.66 × 10^4^ kg. These result in a total of 28.16 × 10^4^ kg, and 65% of which is stemming from concrete material.

**Fig. 16:**
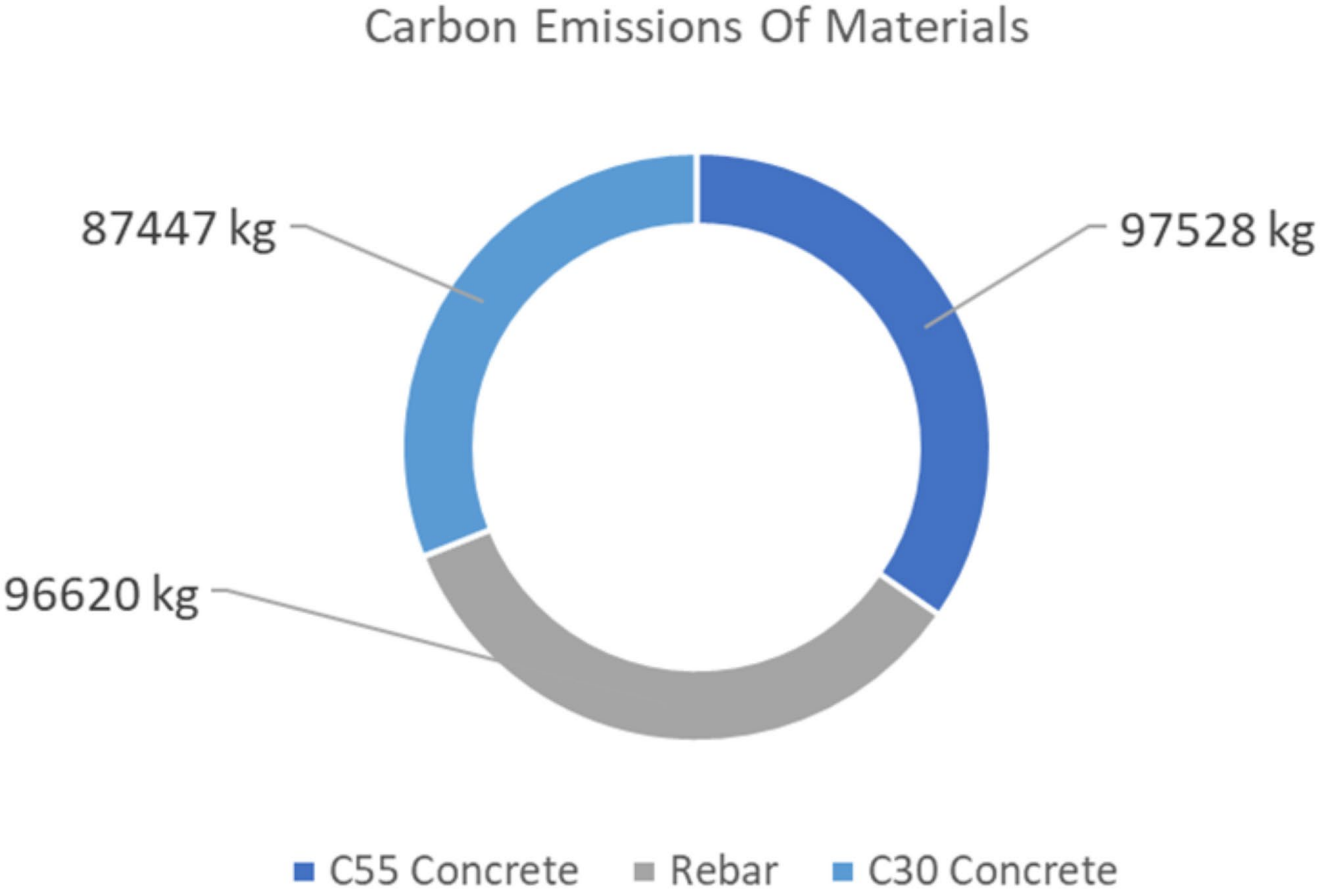
Carbon emissions estimation at different stages of asset lifecycle of the bridge model.

For a more holistic quantification of carbon footprint of the asset, the embodied carbon of the machinery used is also considered. Types of the machinery of each stage of the asset lifecycle are outlined (as shown in Table 4). The predicted energy consumption of each machine is quantified by using values determined from previous analysis, as seen in Table 5. An average usage of 1 h per day of construction and a transit distance of 50 km to site along with the relevant CO_2_e factors (presented in the methodology section), is used to generate a carbon footprint estimation for all the different machineries. Figure 16 shows how the radial bar chart of the carbon emissions distributes all the different stages of the asset lifecycle for the bridge infrastructure, resulting in a total footprint of 444.7 tonnes of carbon, 85% of which is predominantly produced during the construction stage.

**Table 4:** Types of machinery involved during different stages of asset lifecycle.

Machines	Design & planning	Pre-construction	Construction	Operation & maintenance	Demolition
Staff vehicles	1	1	2	1	1
Drilling machine	-	-	2	-	-
Welder	-	-	2	-	-
Bar straightening machine	-	-	1	-	-
Bar cutting machine	-	-	1	-	-
25t crane truck	-	-	1	-	-
200t crane truck	-	-	1	-	-
Concrete mixer	-	-	3	-	-

**Table 5:** Type of machinery and their fuel consumption.

Machine	Consumption	Fuel Type
Staff Vehicles	0.071 L/ km	Fuel Oil
Drilling Machine	29 L/ hour	Diesel
Welder	3.823 kW/ hour	Electricity
Bar Straightening Machine	7.5 kW/ hour	Electricity
Bar Cutting Machine	7.5 kW/ hour	Electricity
25t Crane Truck	76 L/ hour	Diesel
200t Crane Truck	105 L/ hour	Diesel
Concrete Mixer	0.37 L/km	Diesel

## Discussion

In this paper, a conventional 3D BIM model has been further developed to form a digital twin with additional three dimensions (time, cost, and carbon emission schedules) that are able to provide automatic outputs when such a material type is updated. The novel digital twin can simulate the demolition sequence that enables circular economy practices. Validation of the BIM process has been conducted through industry data (e.g. construction schedule and sequence) and expert interviews (by structural design engineers, chief bridge inspectors, bridge maintainers and construction managers at the Department of Highway, Thailand). The scope of this research has not been investigated widely elsewhere. The demolition aspect has been validated partly by asset managers but, due to the lack of recycling specification, only the demolition process and sequences have been validated. The recyclability and reuse after the demolition process has not been fully validated due to the lack of data. Despite an effective approach used for the BIM implementation, the response still lacks sufficient details to be considered for practical use (such as future recycling technologies, reuse potentials, etc.). This is a limitation of the current study where the demolition options are limited to current practial guidelines. However, the digital twin enables the data updating to inform a new decision in the future when new technologies and data become available (e.g. new maintennace regime, new recycling or upcycling approaches, etc.). On this ground, additional information, data and dimensions are simultaneously required to update the DT and better manage the sustainable lifecycle of the asset. The development required can be achieved through performance feedback via the use of sensors and cameras once the asset has been constructed, along with other data inputs or inspection reports from the field staff.

## The application and validation of the proposed BIM model

With respect to bridge applications, a BIM model can be considered at the design stage of a new bridge infrastructure. It can unleash significant benefits for all stages of the asset lifecycle. In this paper, a Level of Development (LOD) of 300 has been considered as a reference, which can adequately create the 6D BIM using the specific size, shape, location, orientation, and quantity of each element of the bridge (for example, material quantities, a decent estimation of cost and carbon footprint)^34^. This enables all stakeholders to obtain a deeper understanding into these key metrics for the whole asset lifetime before the construction. The stakeholders can then take part in decision making more effectively. Intricate details in the design stage of the asset lifecycle can significantly affect latter stages through increasing levels of communication among all stakeholders, allowing for improved collaboration and participation of stakeholders across value chain. This also helps engineers to observe the dynamic response of the real-time information when there is any change or update.

The validation of the proposed 6D BIM model is considered by using a LOD of 300, which is a suitable reference to facilitate accurate estimations of whole-life material costs and embodied carbon. Nevertheless, these estimations can be improved by increasing LOD up to 500, providing additional information to stakeholders. These include element connections, fabrication details and operational components, making a more accurate and holistic idea of the assets’ cost and carbon footprint.

Using software Autodesk Revit for BIM modelling can help designers and engineers to better visualise designs and examine complex structure and components. The components can also be investigated separately from the entire structure, which is useful for sustainability assessment in each stage of the asset lifecycle. In principle, simulations conducted in our study can predict structural integrity and potential faults within the structure, which are required to be repaired during the operation stage of the asset lifecycle. In addition, these potential faults obtained can be negated through either re-design or alteration of the particular components. Alternatively, predictive maintenance rather than reactive or corrective maintenance can be adopted for asset management. This can result in significant savings of cost and raw material^36^.

When the scheduling information is added to a 3-dimensional architectural model, the time-domain analyses of the asset lifecycle of the bridge infrastructure can be investigated. The time-domain results demonstrate that the time schedule of the bridge infrastructure can display critical path and value chain of stakeholders with an accurate estimation of when the bridge will be constructed, when it can be maintained, and when it should be demolished. This will enable credle to credle lifecycle management of the bridge infrastructure, which is a highlight of this study. Also, an effective time schedule can enable any project plans to be completed with the help of sufficient preparation and strategic planning, resulting in a clear roadmap for managing the asset throughout the lifecycle. This can lead to massive productivity derived from clearer preparative milestones that can reduce labour requirements for a given project.

A time schedule for the asset lifecycle helps engineers to minimise conflicts among stakeholders and/or mistakes within construction stage. An additional benefit of having a predefined time schedule for the asset lifecycle is risk identification for the stakeholders. According to a previous study^29^, identifying emerging risks and analysing them at such an early stage allows measures to be implemented, that effectively mitigate the risks before the construction begins. The results in our study exhibit that 85% of carbon emissions are released during the pre-construction and construction stage. Another critical issue of the asset lifecycle management is to consider sustainability. The time schedule can allow all stakeholders to optimise construction times through various construction techniques and options. Alterations to the time schedule can be made (e.g. different manufacturing durations of components made from different materials). This implies that constructors can use prefabricated components or different machinery to totally reduce lead times, leading to a quantified output of carbon emission reduction. The second additional information dimension in the digital twin is the cost schedule. A cost schedule allows the stakeholders to manage cost during both the design, construction and maintenance stages. The results depict that 66.4% of the total cost of the asset throughout its lifecycle results from the construction stage, which is stemmed from relatively high raw material cost. Our results offer a certain promise to further utilisation of digital twin for stakeholders. The cost can be adjusted through different material usage, and different material options will have different unit costs and can therefore be compared to indicate the changes affecting the budgets and sustainability goals. It is clear that the digital twin can provide instantaneous response to observe whether the material cost is aligned with the existing budget policy.

Reportedly, raw materials sourced from clean and green factories are currently more expensive than traditional manufacturing approaches that predominantly use fossil fuels. Therefore, the cost scheduling is imperative to ensure that designers remain within approved budgets. It will also hold engineers accountable in reaching their sustainability goals. A limitation of this study is the assumption that the costs of the same items remain constant in the analysis. In fact, the cost cashflows could be different in any particular year. However, due to limited data, we assume that the costs are relatively similar across the lifetime. In the future, better data collection will help the digital twin predict and quantify economic impact much more precisely.

The final dimension of the digital twin considered for the sustainability of the bridge infrastructure is the carbon emission schedule. The ability to accurately quantify carbon emissions enables stakeholders to consider various sensitivity metrics (e.g. carbon credit) for low carbon outputs. It is evident that the raw materials and the machinery used to construct the bridge infrastructure are responsible for 85% of carbon emissions during the entire lifecycle of the asset. This points out that the construction stage of the lifecycle is the most crucial stage in defining the sustainability of the infrastructure.

In addition to carbon emissions during the construction stage, engineers and constructors can potentially reduce the emissions by using low carbon materials (such as low carbon concrete^37^) or machineries powered by renewable energy (such as sustainably generated electricity). The results show that most of the machineries used in the construction stage are powered by diesel. Unlike construction machineries using electricity power, the construction process with the raw materials would significantly reduce the carbon emissions displayed in this study. Thus, it is necessary to consider the energy consumption of the machinery used in a project. On the other hand, it is difficult to quantify exact operational time requirements for different machinery causing their carbon emissions. The improvement of machinery choices for construction should thus be considered in order to make better estimations of carbon emissions.

Consequently, the digital twin for the bridge infrastructure can be a representative promising a centralised information platform in which design and maintenance can be shared, altered, and tested to remain in line with stakeholders’ requirements. This creates a favorable environment for all stakeholders to collaborate and exchange information to mitigate risks and develop improved participatory co-designs prior to construction for a sustainable lifecycle from cradle to cradle. The demolition planning simulation capability within the digital twin can underpin the EU’s Circular Economy Action Plan. This will help construction industry and stakeholders to adopt and implement science-based targets across value chain towards net zero swiftly.

This paper highlights a rudimentary method of 6D BIM implementation towards a ditigal twin for cradle to cradle lifecycle management. Although there is limited literature within this area, the results have shown significant opportunities for further research. Our future research will focus on the integrity and accuracy of longer-term datasets within the 6D BIM, which can help engineers develop adaptative lifecycle management for other bridge infrastructures. Note that, for more complicated 6D BIM models, specific training for engineers and end-users will be required to ensure the functionality of these models, which can also incite extra project costs.

## Conclusions

In this paper, an innovative digital twin used for sustainable lifecycle assessment of a road bridge is presented. This study displays a clear, reproduceable method for which a digital twin can be implemented for cradle-to-cradle lifecycle simulations. Although this study focuses on a particular bridge design, this method can be transferred for any type of bridge infrastructures. Unprecedentedly, a digital twin has been generated during pre- or post-construction of the asset lifecycle, although it could actually produce significantly greater sustainability benefits if it can be implemented during the design stage of the lifecycle. This paper demonstrates how a digital twin approach can minimise time-consuming tasks and re-work during the initial stages of the bridge infrastructure lifecycle. During these initial stages, the digital twin also enables all stakeholders to accurately visualise the infrastructure prior to actual construction. Most notably, the stakeholders are able to quantify how sustainable the asset will be through its entire lifetime considering the carbon emission outputs that the digital twin is able to provide. This study provides new evidence underpinning the importance of digital twin as a process framework for cradle-to-cradle sustainable lifecycle management of bridge infrastructure. The research outcome derived from this study is currently being implemented in practice by Thailand’s Department of Highways.

Our findings reveal some new insights:

- The major contributor to carbon emissions and costs is the construction stage of the asset lifecycle, which is predominantly derived from raw materials. This has a follow-on effect on the end-of-life management of such materials.
- Surprisingly, our digital twin can minimise design and re-work, mitigate risk, and real-time update of design changes for all stakeholders, resulting in real-time decision-making information on carbon emissions, costs, as well as time schedules.
- The demolition process and planning by the digital twin can increase circular economy implementation. It is evident that our digital twin can be a useful tool for sustainable lifecycle management of bridge infrastructures from cradle to cradle.
- Nevertheless, the industry use of 6D-BIM-based digital twin approach is still limited for the lifecycle management of complex bridge infrastructure, as it is primarily used for standard building and architectural requirements. For example, the input for rebars and the modelling of complicated connections are currently difficult to implement for lifecycle assessment. This is due to the topological complexity of the sub-components and connections. Thus, the advanced development of the BIM package is considerably required in order to easily address this issue and be much more compatible with the modelling of complex infrastructures.

In summary, the application of digital twin shows immense promises for all stakeholders for participatory co-design and co-planning of sustainable lifecycle management. Indeed, it is a relatively new approach to sustainable lifecycle management, but the potential is clear, and we can expect that digital twins will become an essential tool for the infrastructure design industry globally.

## Data Availability

The data that support the findings of this study are available from the corresponding author upon reasonable request.
