# Author Correction: Digital twin-driven strategic demolition plan for circular asset management of bridge infrastructures

**DOI:** 10.1038/s41598-025-98324-1

**Published:** 2025-04-28

**Authors:** Sakdirat Kaewunruen, Connor O’Neill, Pasakorn Sengsri

**Affiliations:** 1 Department of Civil Engineering, School of Engineering, University of Birmingham, Edgbaston, B152TT UK; 2 Digital Asset Management, AtkinsRéalis, Birmingham, UK; 3 Department of Highways, Bangkok, Thailand

Correction to: *Scientific Reports* 10.1038/s41598-025-94117-8, published online March 2025

The original version of this Article contained an error in the spelling of the author Connor O’Neill

which was incorrectly given as Connor O’Nell.

The original Article has been corrected.

